# Horses give functionally relevant responses to human facial expressions of emotion: a response to Schmoll (2016)

**DOI:** 10.1098/rsbl.2016.0549

**Published:** 2016-09

**Authors:** Amy Victoria Smith, Leanne Proops, Kate Grounds, Jennifer Wathan, Karen McComb

**Affiliations:** Mammal Vocal Communication and Cognition Research Group, School of Psychology, University of Sussex, Brighton BN1 9QH, UK

Our paper demonstrates that horses show a quicker increase in heart rate when presented with photographic stimuli depicting angry versus happy human faces. We also use lateralized looking at each stimulus as a means of investigating how the image is perceived and found a strong left gaze/right hemisphere bias to angry stimuli but no significant lateralized response to happy or significant difference between responses to angry and happy. While we accept that a more extensive exploration and discussion of the results would have been useful, our original findings still stand.

Schmoll [1] voices two main concerns: the inclusion of three very short latency-to-response values in the heart-rate data and our interpretation of the lateralization results. Dealing first with the heart-rate data, Schmoll raises a useful point about whether very short latency values (less than 1 s) can be attributed to an immediate response to the stimuli and we are pleased to explore this possibility. Our original analysis included a few seconds while the stimulus photo was being turned towards the subject and was only partly visible. To ensure that our latency-to-maximum heart-rate values best reflect direct responses to stimuli, we re-analysed our heart-rate data with a more conservative start point—the heartbeat immediately before the stimulus fully faced the subject. In this re-analysis, two horses (Rose: happy trial; Willsie: angry trial) whose heart rates did not increase from the test start were excluded. With this start point, heart rate still increased faster towards angry photographs compared with happy (angry: mdn = 14.7 s, happy: mdn = 29 s; Wilcoxon test: *z* = −2.39, *p* = 0.015; figure 1). Only one subject has a response latency under 5 s (Jack = 1.6). While our on-going work suggests horses' heart rates can respond within 1.6 s, even if Jack is removed the effect remains (angry: mdn = 17.7 s, happy: mdn = 29.10 s; *z* = −2.17, *p* = 0.03).

**Figure 1.**
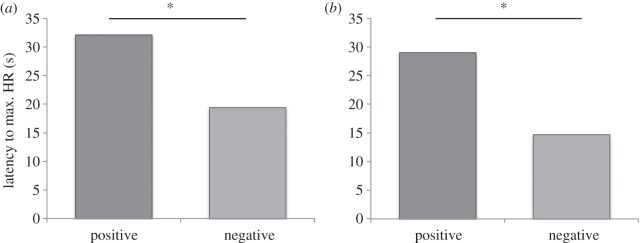
Median latency to maximum heart-rate comparison (*a*) original data (*b*) re-analysed data, *p < 0.05.

Thus on the basis of heart-rate data alone, horses discriminate between angry and happy human expressions. On Schmoll's second point, we accept that our lateralization results cannot confirm a between-groups difference and for most of the paper we discuss the lateralization results independently for the two emotions. Our discussion [2] opens: “The behavioural and physiological results reported here support the hypothesis that horses are able to recognize and respond in a functionally relevant way to heterospecific (human) facial expressions of anger”, and we go on to explore reasons for a lack of lateralized response to the positive expressions. Furthermore, we ourselves present a *t*-test showing a non-significant difference between the stimuli in the legend of figure 2. Schmoll's mixed model concurs with our *t*-test and both are appropriate analyses. We agree it would have been desirable to give greater prominence to this result, and the line of our conclusion that Schmoll focuses on somewhat confounds the behavioural and physiological results, combining several lines of evidence all pointing in one direction (heart rate, lateralization, stress-related behaviours), rather than making clear the different components of the argument. We expand our discussion below, adding a control that supports our original interpretation.

**Figure 2.**
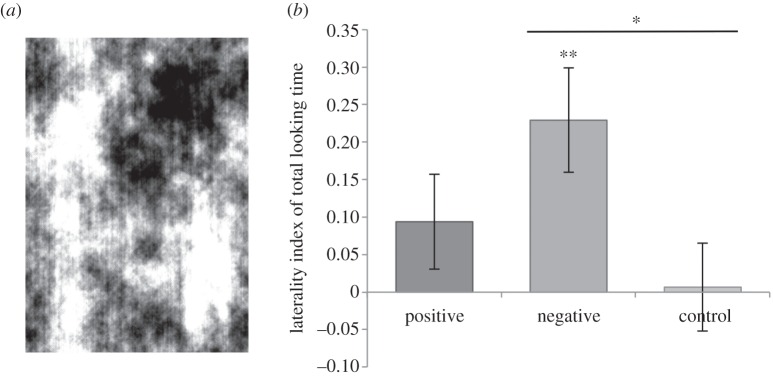
(*a*) Phase-scrambled control stimuli (*b*) Mean laterality index by condition including additional control (±1 s.e.m.), *p < 0.05 (negative compared to control) **p < 0.01 (negative compared to zero).

While comparing results to chance may not allow for direct comparisons between groups, it answers an important and distinct question about whether the response to each emotion is lateralized and is an accepted method for analysing laterality indices [3]. In our study, the left gaze bias suggests that responses to angry faces were strongly lateralized in the right hemisphere (one sample *t*-test on laterality index: *t*_27_ = 3.49, *p* = 0.002), concurring with several other variables (heart rate and stress-related behaviours) in indicating that horses perceive the expression as negatively valenced. This is consistent with a large body of evidence showing negative emotional stimuli are preferentially processed in the right hemisphere [4]. Our results also revealed no evidence of lateralized gaze responses to positive stimuli (*t*_27_ = 1.48, *p* = 0.15). The same pattern of responses to human emotional expressions is seen in dogs [3], and these findings collectively point to interesting avenues for future research.

Schmoll also suggests that our results could arise from a stressful test set-up or the position of the experimenter biasing the response. We can address these points by comparing our laterality data with a control where, using the same presentation protocol, horses viewed phase-scrambled images of horse facial photographs (figure 2; [3]). Twenty-eight test subjects in our paper were compared with 28 independent control subjects (21 from a previous study; seven additional to replace repeated subjects and increase *N* to 28)*.* Here, the responses of the control group were not significantly lateralized (*M* = 0.007, s.e.m. = 0.06, *t*_27_ = 0.12, *p* = 0.91). Moreover, there was a significant difference between the three conditions when compared directly using a linear mixed effects model with *emotion* (happy/angry/control) as a fixed effect and subject as a random effect (*t*_84_ = 3.32, *p* = 0.041). In post hoc *t*-tests (Bonferroni corrected), the responses of the control subjects differed significantly from those of test subjects to the angry (*t*_54_ = −2.53, *p* = 0.02, figure 2) but not to the happy stimuli (*t*_54_ = −1.01, *p* = 0.64). As well as providing an extra control for our original presentations, these findings address Schmoll's remaining points—that the left gaze bias may have resulted from a stressful test situation (a possibility we ourselves considered in the discussion), or from the handler standing on the horse's left (a standard handling position for horses). As the handler's position was consistent and any test-related stress was also present in our control trials, neither appears to have driven the behavioural responses.

To summarize, our paper presents multiple strands of evidence (heart rate, laterality and displacement behaviours) that support our argument and are in line with findings in other species. While we acknowledge we could have been clearer in one sentence of the conclusion, and given more prominence to the *t*-test results, the analyses and interpretations are not fundamentally flawed as Schmoll describes. When we re-examine our heart-rate data taking into account Schmoll's point about short latencies and add an additional control on laterality effects, it strengthens our original findings and interpretations. Consequently, we can reaffirm that our results demonstrate that horses give functionally relevant responses to human facial expressions of emotion as initially reported.

## References

[1] Schmoll T 2016 Can horses read emotional cues from human faces? Re-analysis of Smith *et al*. (2016). Biol. Lett. 20160201 (10.1098/rsbl.2016.0201) 27624794 PMC5046913

[2] Smith AV, Proops L, Grounds K, Wathan J, McComb K 2016 Functionally relevant responses to human facial expressions of emotion in the domestic horse (*Equus caballus*). Biol. Lett. 12, 20150907 (10.1098/rsbl.2015.0907) 26864784 PMC4780548

[3] Racca A, Guo K, Meints K, Mills DS 2012 Reading faces: differential lateral gaze bias in processing canine and human facial expressions in dogs and 4-year-old children. PLoS ONE 7, e36076 (10.1371/journal.pone.0036076) 22558335 PMC3338636

[4] Rogers LJ 2002 Lateralization in vertebrates: its early evolution, general pattern, and development. Adv. Stud. Behav. 31, 107–161. (10.1016/S0065-3454(02)80007-9)

[5] Wathan J, McComb K 2014 The eyes and ears are visual indicators of attention in domestic horses. Curr. *Biol.* 15, 677–679. (10.1016/j.cub.2014.06.023) PMC4123162 25093554

